# SUMOylation targets O-GlcNAcase to chaperone-mediated autophagy

**DOI:** 10.1016/j.jbc.2025.110314

**Published:** 2025-05-29

**Authors:** Sheng Yan, Aiyun Yuan, Guangcan Shao, Wen Zhou, Xin Xu, Meng-Qiu Dong, Xiaoqian Liu, Jing Li

**Affiliations:** 1 Beijing Key Laboratory of DNA Damage Response and College of Life Sciences, Capital Normal University, Beijing, China; 2 National Institute of Biological Sciences, Beijing, China; 3 College of Chemistry and Molecular Engineering, Peking University, Beijing, China; 4 Department of Obstetrics and Gynecology, Beijing Friendship Hospital Affiliated to Capital Medical University, Beijing, China; 5 State Key Laboratory of Microbial Technology, Shandong University, Qingdao, China

**Keywords:** O-GlcNAcase, SUMOylation, Hsc70, chaperone-mediated autophagy (CMA), YEATS2

## Abstract

O-GlcNAcase (OGA) is the sole eraser for the intracellular O-GlcNAc. OGA has many roles in distinct biological processes, such as cancer and embryonic stem cells, but its precise regulatory mechanism is far from being understood. Herein, we studied the small ubiquitin-like modifier (SUMO) modification of OGA and found that OGA is SUMOylated at K358. SUMOylation targets OGA to the chaperone-mediated autophagy (CMA) pathway, which shunts client proteins to the lysosome for degradation. We demonstrate that SUMOylation increases the association between OGA and the heat shock cognate protein 70 (HSC70), the CMA chaperone, and facilitates OGA further degradation. We further mapped a SUMO-interacting motif (SIM) (VLIFD, aa. 195–199) on HSC70. Notably, HSC70-SIM is essential for affinity with other CMA client proteins, such as pyruvate kinase M2. We thus posit that the SIM of HSC70 binds SUMOylated client proteins in a lock-and-key manner to confer substrate selectivity during CMA. To further test our hypothesis, we used label-free quantitative mass spectrometry to study the HSC70-SIM mutant interactome and generated a proteome-wide SUMO-mediated CMA client pool. We then validated this model by studying YEATS domain-containing two from the protein pool and demonstrated that YEATS domain-containing two is SUMOylated at K592, targeting it to CMA. Our work uncovers the SUMO–SIM interaction as a fundamental mechanism governing CMA substrate selectivity and identifies a potential CMA client proteome to deepen our understanding of its pathophysiological relevance.

The intracellular O-GlcNAc modification is mediated by a pair of regulators: O-GlcNAc transferase (OGT), the writer, and O-GlcNAcase (OGA), the eraser (1). Over the past 4 decades, research has focused on elucidating O-GlcNAc substrates and their biological functions. Over 7000 of these substrates have been identified. Leveraging chemical biology and mass spectrometry (MS), the O-GlcNAcome continues to increase. Although a consensus sequon on O-GlcNAc substrates is still elusive, structural studies reveal that the 5 N-terminal tetratricopeptide repeat domains are indispensable for proper OGT localization and protein–protein interaction (2, 3).

Relatively less is understood about OGA. Originally OGA was thought to possess acetyltransferase activity, due to its C-terminal pseudo histone-acetyltransferase domain. However, OGA is later found not to be able to bind acetyl-coA, and therefore devoid of acetyltransferase activity. But under high glucose, OGA could facilitate pyruvate kinase M2 (PKM2) acetylation and upregulate PKM2 O-GlcNAcylation (4). During DNA damage response, OGA is recruited to damage sites, in a manner dependent on the C-terminal pseudo-histone-acetyltransferase domain. OGA recruitment reverses O-GlcNAcylation catalyzed by OGT and is required for subsequent nonhomologous end joining (5, 6). OGA is highly expressed in naïve human embryonic stem cells and is essential for naïve pluripotency maintenance (7). It is more complicated in other model organisms, as OGA is yet to be identified in plants, and OGA is not essential in *Drosophila* (8).

How OGA is regulated posttranslationally is even more unclear. As early as 2006, OGA was found to associate with OGT (9), and later, OGA was found to be O-GlcNAcylated at S405 by MS (10), which is also confirmed in cryo-EM structural studies (3), and it modulates OGA stability (11). At the single amino acid level, the cancer-derived OGA-S652F mutant prefers substrates with Pro at the +2 position to the O-GlcNAc sites, such as PDZ and LIM domain 7, leading to p53 dysregulation and cell malignancy (12).

In this work, we studied the small ubiquitin-like modifier (SUMO) modification on OGA. We first confirmed that OGA is SUMOylated at K358, as revealed in previous proteomic screens (13). Interestingly, K358 resides in the middle of the pentapeptide (KFERQ) motif that is characteristic of the chaperone-mediated autophagy (CMA) client proteins recognized by heat shock cognate protein 70 (HSC70) (14). CMA is a selective autophagic process, in which HSC70 binds KFERQ-bearing client proteins and delivers them to lysosome-associated membrane protein type 2a (Lamp2a), a lysosome receptor. Thereafter, the client proteins are degraded in the lysosome. Proper CMA function has been linked to glucose and lipid metabolism, DNA repair and other biological processes, besides protein quality control.

We demonstrate that SUMOylated OGA binds the SUMO-interacting motif (SIM) of HSC70, targeting OGA to CMA-mediated degradation. We further utilized label-free quantitative MS to demonstrate that HSC70-SIM is pivotal to recognizing SUMOylated CMA client proteins, such as YEATS domain-containing 2 (YEATS2). Similar to a lock and key, SUMO-SIM binding thus confers CMA substrate selectivity. Our work not only demonstrates that OGA is subject to CMA-mediated degradation, but also provides a potential CMA client protein resource that will contribute to our complete understanding of CMA in physiological settings.

## Results

### Ubc9 interacts with OGA

**Figure 1:**
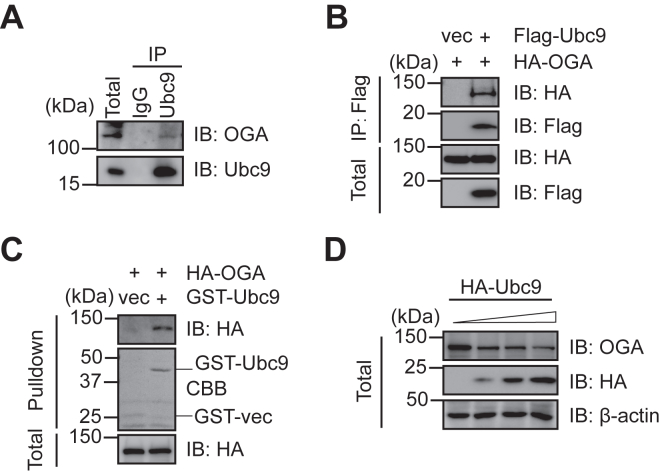
**Ubc9 interacts with OGA.***A,* coimmunoprecipitation between endogenous Ubc9 and OGA. *B*, cells were transfected with Flag-Ubc9 and HA-OGA plasmids. The lysates were immunoprecipitated with anti-Flag antibodies and immunoblotted with anti-HA and anti-Flag antibodies as indicated. *C*, cells were transfected with HA-OGA plasmids. Recombinant GST-Ubc9 proteins were incubated with the cellular lysates, and GST pull-down experiments were carried out. *D*, cells were transfected with 2 μg, 5 μg, and 10 μg HA-Ubc9 plasmids, respectively. Cellular lysates were collected and subject to immunoblotting assays. OGA, O-GlcNAcase; GST, glutathione-*S*-transferase.

We sought to identify potential posttranslational modifications (PTMs) on OGA. A previous proteomic screen demonstrated that OGA is SUMOylated at K358 (www.phosphosite.org) (13), prompting us to examine whether OGA possesses SUMO modification. We first examined the interaction between OGA and Ubc9, the sole SUMO-conjugating enzyme (15). Cell lysates were immunoprecipitated (IPed) with anti-Ubc9 antibodies, and OGA was detected in the immunoprecipitates (Fig. 1*A*). We also examined the interaction between overproduced proteins (Fig. 1*B*). Cells were transfected with Flag-Ubc9 and hemagglutinin (HA)-OGA plasmids, and the lysates were IPed with anti-Flag antibodies. The results showed that HA-OGA co-immunoprecipitates with Flag-Ubc9. Recombinant glutathione-*S*-transferase (GST)-Ubc9 proteins were also utilized. Cells were transfected with HA-OGA, and the cellular extracts were incubated with recombinant GST-Ubc9 proteins (Fig. 1*C*). These results suggest that Ubc9 and OGA associate with each other.

We sought to examine whether Ubc9 regulates OGA protein levels. When Ubc9 is overexpressed in a dosage-dependent manner, it significantly attenuates the level of endogenous OGA proteins (Fig. 1*D*). These results demonstrate that Ubc9 negatively regulates OGA stability.

### OGA is subject to SUMO1 modification at K358

**Figure 2:**
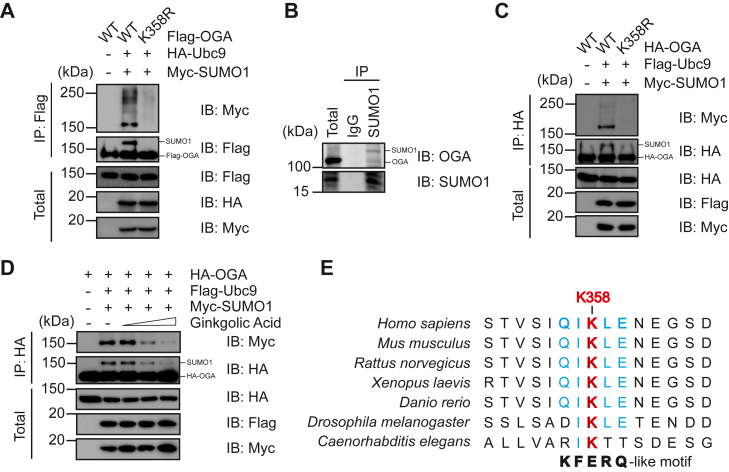
**OGA is subject to SUMO1 modification at K358.** A, HEK-293T cells were transfected with Flag-OGA-WT, FLAG-OGA-K358R, HA-Ubc9 and Myc-SUMO1 plasmids, and then the lysates were immunoprecipitated and immunoblotted with the antibodies indicated. *B*, coimmunoprecipitation between endogenous SUMO1 and OGA. *C*, HEK-293T cells were transfected with HA-OGA-WT, HA-OGA-K358R, Flag-Ubc9, and Myc-SUMO1 plasmids. *D*, HEK-293T cells were transfected with HA-OGA, Flag-Ubc9, and Myc-SUMO1, and then the cells were treated with ginkgolic acid (GA) at concentrations of 20 μM, 50 μM, and 100 μM, respectively, for 24 h. *E,* OGA K358 is conserved in multiple species, and it sits in the heart of a KFERQ motif of chaperone-mediated autophagy (CMA) client proteins. OGA, O-GlcNAcase; GST, glutathione-*S*-transferase; SUMO, small ubiquitin-like modifier.

We sought to validate whether OGA is SUMOylated at K358 (www.phosphosite.org) (13). Cells were transfected with Flag-OGA, HA-Ubc9, and Myc-SUMO1 plasmids. We observed a slower migrating band detected by the anti-Flag antibody in cells cotransfected with Myc-SUMO1 plasmids, but not in the control (Fig. 2*A*) or SUMO2-transfected cells (data not shown). We constructed the OGA-K358R mutant, and it abolished the SUMO1 modification of OGA (Fig. 2*A*), suggesting that K358R is indeed the SUMO site of OGA. SUMOylation on endogenous OGA without overexpression of the SUMO machinery was examined (Fig. 2*B*), and OGA was identified in the anti-SUMO1 immunoprecipitates. We also constructed HA-OGA-K358R and again the SUMO band was abolished in the mutant (Fig. 2*C*). These findings indicate that K358 is a major SUMO1 site of OGA.

To further validate that the band we observed is specifically the SUMO signal, we treated the cells with ginkgolic acid (GA), an inhibitor of SUMOylation (16). The results showed that the SUMO bands weakened with increasing GA dosages (Fig. 2*D*). Moreover, sequence alignment demonstrates that OGA K358 is conserved in various organisms (Fig. 2*E*). Taken together, OGA is SUMO1-modified at K358.

### SUMOylation targets OGA to CMA

In a previous investigation of OGA function, Hsc70 was among the top hits in the OGA interactome (5). And Figure 2*E* demonstrated that K358 resides in a KFERQ motif characteristic of CMA client proteins (14). These clues prompted us to examine whether OGA is subject to CMA.

**Figure 3:**
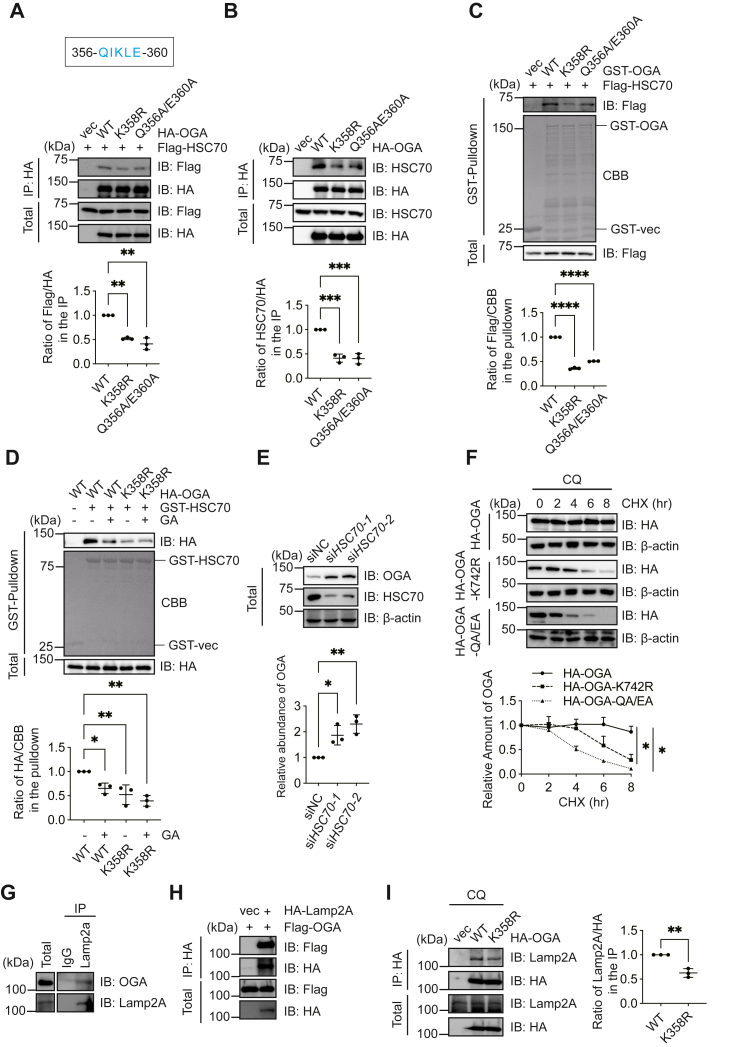
**SUMOylation targets OGA to chaperone-mediated autophagy.***A,* HEK293T cells were transfected with HA-OGA-WT, HA-OGA-K358R, HA-OGA-Q356AE360A, Flag-HSC70 plasmids, and then the lysates were immunoprecipitated and immunoblotted with the antibodies indicated. The ratio of Flag/HA in the IP was quantified and shown in the *bottom panel*. *B*, HEK293T cells were transfected with HA-OGA-WT, HA-OGA-K358R, HA-OGA-Q356A/E360A plasmids, and then the lysates were immunoprecipitated and immunoblotted with the antibodies indicated. The ratio of HSC70/HA in the IP was quantified and shown in the *bottom panel*. *C*, HEK-293T cells were transfected with Flag-HSC70 plasmids. Recombinant GST-OGA-WT, GST-OGA-K358R, and GST-OGA-Q356A/E360A proteins were incubated with the cellular lysates, and GST pull-down experiments were carried out. The quantitation was shown in the *bottom panel*. *D*, HEK293T cells were transfected and treated with 100 μM GA for 24 h. Recombinant GST-HSC70 proteins were incubated with the cellular lysates, and GST pull-down experiments were carried out. The quantitation was shown in the *bottom panel*. *E*, cells were transfected with two siRNAs targeting *HSC70*. *F*, cycloheximide (CHX) pulse-chase assays. Cells were transfected with HA-OGA-WT, HA-OGA-K358R, or HA-OGA-Q356A/E360A plasmids, and then treated with chloroquine (CQ) and CHX for different durations. *G*, coimmunoprecipitation between endogenous Lamp2a and OGA. *H*, cells were transfected with HA-Lamp2a and Flag-OGA. *I*, HEK293T cells were transfected with HA-vec, HA-OGA-WT and HA-OGA-K358R plasmids and treated with 10 μM CQ for 3 h. Quantitation in (*A*–*F*) was carried out with one-way ANOVA. Quantitation in (*I*) was carried out with Student's *t* test. ∗*p* < 0.05; ∗∗*p* < 0.01; ∗∗∗*p* < 0.001; and ∗∗∗∗*p* < 0.0001. OGA, O-GlcNAcase; GST, glutathione-*S*-transferase; IP, immunoprecipitation; GA, ginkgolic acid; CHX, cycloheximide; Lamp2a, lysosome-associated membrane protein type 2a; HSC70, heat shock cognate protein 70.

We generated OGA-Q356A/E360A, the mutant of the KFERQ motif, and observed that both OGA-K358R and OGA-Q356A/E360A mutants bound less HSC70 in cells (Fig. 3*A*), which was also observed in the interaction with endogenous HSC70 (Fig. 3*B*). We then used the GST pull-down assay. Consistently, recombinant OGA-K358R and OGA-Q356A/E360A proteins pulled down less Hsc70 (Fig. 3*C*). And *vice versa*, recombinant GST-Hsc70 proteins pulled down less HA-OGA-K358R and OGA-Q356A/E360A (Fig. 3*D*), suggesting that 356-QIKLE-360 is responsible for the association between Hsc70 and OGA. To determine if the SUMO1 modification of OGA affects its interaction with HSC70, HEK-293T cells were transfected with OGA-WT or OGA-K358R mutants, and then treated with GA. The interaction between OGA-WT and GST-HSC70 was significantly reduced after GA treatment, but there were no significant differences in the OGA-K358R mutant after GA treatment (Fig. 3*D*). Overall, the SUMO1 modification of OGA on K358 facilitates its interaction with HSC70.

To further analyze whether OGA could be degraded through the lysosome, we knocked down HSC70 with two independent si*HSC70* oligos. In HSC70 knockdown cells, we observed more OGA accumulation (Fig. 3*E*). We then sought to examine whether SUMOylation targets OGA to the lysosome by using the lysosome inhibitor chloroquine (CQ). As shown in Figure 3*F*, cells were incubated with CQ and pretreated with cycloheximide (CHX) (Fig. 3*F*). While this treatment induced a marked decrease in OGA-K358R and OGA-Q356A/E360A protein stability, OGA-WT proteins remained stable (Fig. 3*F*). We further used a proteasome inhibitor MG132 in the CHX assay (Fig. S1). MG132 prevents K742R and QA/EA degradation but not WT degradation, indicating degradation of OGA-K742R and OGA-QA/EA occur through the proteasome (Fig. S1). Taken together, the results showed that OGA is degraded *via* both the ubiquitin–proteasome pathway and CMA, and the SUMOylation mutant K742R and the Q356A/E360A mutant are mainly degraded *via* the ubiquitination pathway.

We further tested the affinity between OGA and Lamp2a, the CMA receptor. As illustrated in Figure 3*G*, Lamp2a interacts with endogenous OGA. Upon overexpression, HA-Lamp2a interacts with Flag-OGA (Fig. 3*H*). However, the K358R mutant showed reduced affinity with Lamp2a (Fig. 3*I*). These data indicate that SUMOylation increases the interaction of OGA with HSC70 and Lamp2A and targets OGA for CMA-mediated degradation.

### HSC70 contains a SIM required for CMA client protein binding

**Figure 4:**
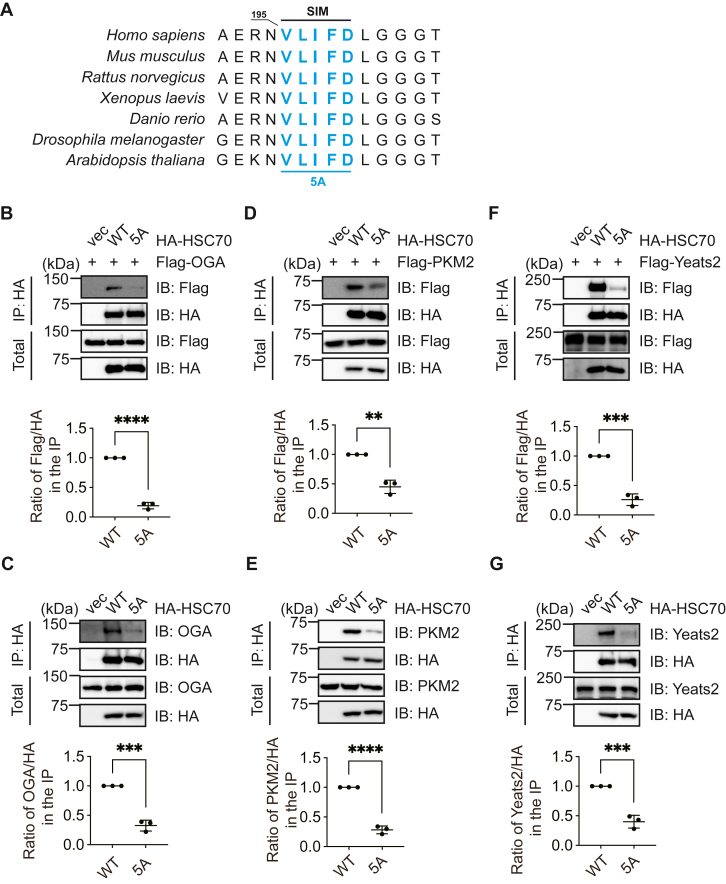
**HSC70 contains a SUMO-interacting motif required to bind client proteins in chaperone-mediated autophagy**. *A,* alignment of HSC70 regions containing the SUMO-interacting motif (SIM) from multiple species. *B*, HEK293T cells were transfected with HA-HSC70, HA-HSC70-V195A/L196A/I197A/F198A/D199A (5A) and Flag-OGA plasmids, and then the lysates were immunoprecipitated and immunoblotted with the anti-HA and anti-Flag antibodies as indicated. The ratio of Flag/HA in the IP was quantified and shown in the *bottom panel*. *C*, HEK293T cells were transfected with HA-HSC70 and HA-HSC70-5A plasmids, and then the lysates were immunoprecipitated and immunoblotted with the anti-HA and anti-OGA antibodies as indicated. The ratio of OGA/HA in the IP was quantified and shown in the *bottom panel*. *D*, HEK293T cells were transfected with HA-HSC70, HA-HSC70-5A, and Flag-PKM2 plasmids, and then the lysates were immunoprecipitated and immunoblotted with the anti-HA and anti-Flag antibodies as indicated. The ratio of Flag/HA in the IP was quantified and shown in the *bottom panel*. *E*, HEK293T cells were transfected with HA-HSC70, HA-HSC70-5A plasmids, and then the lysates were immunoprecipitated and immunoblotted with the anti-HA and anti-PKM2 antibodies as indicated. *F*, HEK-293T cells were transfected with HA-HSC70, HA-HSC70-5A, and Flag-Yeats2 plasmids, and then the lysates were immunoprecipitated and immunoblotted with the anti-HA and anti-Flag antibodies as indicated. The ratio of Flag/HA in the IP was quantified and shown in the *bottom panel*. *G*, HEK293T cells were transfected with HA-HSC70 and HA-HSC70-5A plasmids, and then the lysates were immunoprecipitated and immunoblotted with the anti-HA and anti-Yeats2 antibodies as indicated. The quantitation of (*B*–*G*) was carried out with Student's *t* test. ∗∗*p* < 0.01; ∗∗∗*p* < 0.001; and ∗∗∗∗*p* < 0.0001. OGA, O-GlcNAcase; GST, glutathione-*S*-transferase; HSC70, heat shock cognate protein 70; IP, immunoprecipitation; Lamp2a, lysosome-associated membrane protein type 2a; SUMO, small ubiquitin-like modifier; YEATS2, YEATS domain-containing 2.

As SUMOylation is known to associate with the SIM, we wondered whether Hsc70 contains a SIM that is essential for interacting with OGA. Using a bioinformatics tool (https://sumo.biocuckoo.cn), we found a putative SIM in Hsc70 (^195^VLIFD^199^), which is conserved in various species (Fig. 4*A*). We constructed the Hsc70-VLIFD-AAAAA (5A) mutant accordingly and found that Hsc70-5A significantly attenuated association with both overproduced and endogenous OGA (Fig. 4, *B* and *C*).

This observation raised an intriguing hypothesis: could HSC70-SIM mediate CMA client protein interaction through a SUMO-SIM binding mechanism? PKM2 has been reported to be modified by SUMOylation and degraded *via* the CMA pathway (17, 18). We examined the interaction between Hsc70 and PKM2 and found that HA-HSC70-5A bound less Flag-PKM2 and endogenous PKM2 in cells (Fig. 4, *D* and *E*), suggesting that a SUMO–SIM interaction underlies CMA client recognition.

### Label-free quantitative MS identifies potential CMA client proteins, including YEATS2

We wondered whether this SUMO-SIM locking mechanism could be applied to the whole proteome, so we performed a label-free quantitative MS analysis of the HA-HSC70-WT and HA-HSC70-5A interactome to obtain a potential list (Table S1), among which is YEATS2. YEATS2 has not been reported to undergo SUMOylation or degradation *via* the CMA pathway. We examined the interaction between YEATS2 and HA-HSC70. Not only does YEATS2 interact with Hsc70, but also Hsc70-5A decreases interaction with both overproduced and endogenous YEATS2 (Fig. 4, *F* and *G*).

**Figure 5:**
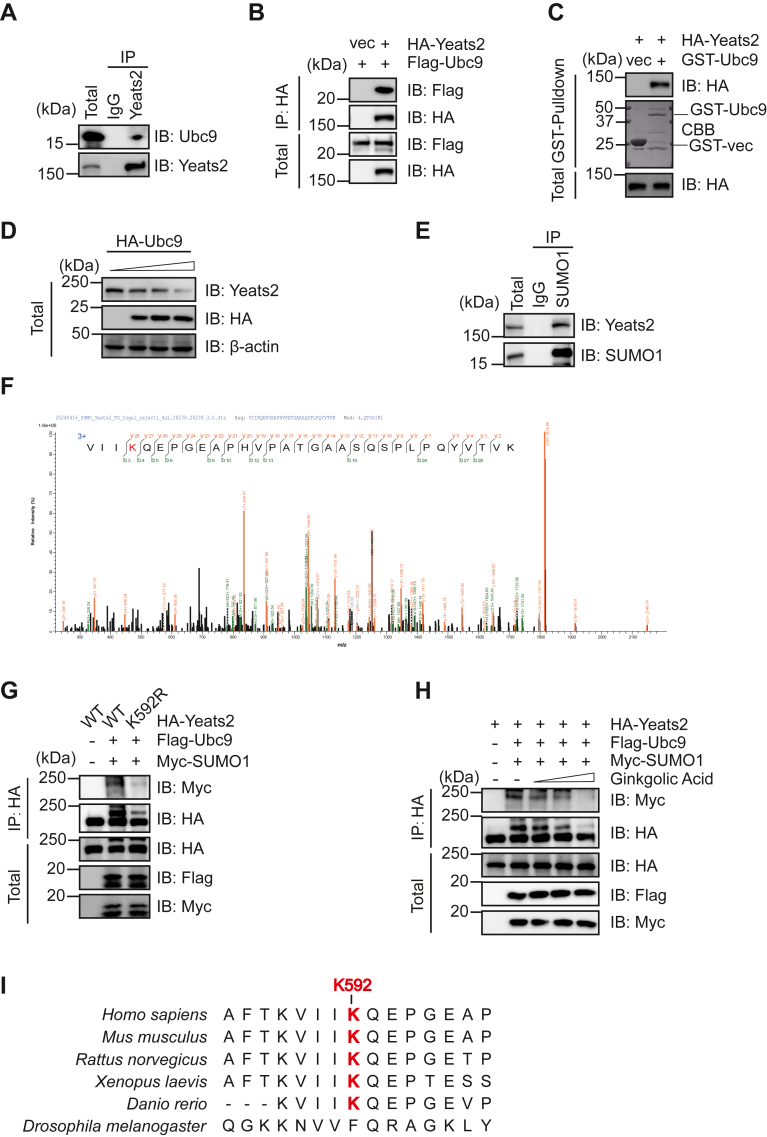
**YEATS2 is subject to SUMO1 modification at K592.***A,* co-IP between endogenous YEATS2 and Ubc9. *B,* cells were transfected with HA-YEATS2 and Flag-Ubc9 plasmids. *C,* cells were transfected with HA-YEATS2 plasmids. Recombinant GST-Ubc9 proteins were incubated with cellular lysates, and GST pull-down experiments were carried out. *D*, cells were transfected with 2 μg, 5 μg, and 10 μg HA-Ubc9 plasmids, respectively. Cellular lysates were collected and subject to immunoblotting assays. *E*, co-IP between endogenous SUMO1 and YEATS2. *F*, cells were transfected with HA-YEATS2, Flag-Ubc9, and Myc-SUMO1. Anti-HA immunoprecipitates were subject to mass spectrometry analysis. *G*, HEK-93T cells were transfected with HA-YEATS2-WT, HA-YEATS2-K592R, Flag-Ubc9, and Myc-SUMO1. *H*, HEK293T cells were transfected with HA-YEATS2, Flag-Ubc9, and Myc-SUMO1, and then the cells were treated with GA at concentrations of 20 μM, 50 μM, and 100 μM, respectively, for 24 h. *I,* YEATS2 K592 is conserved in multiple species. GST, glutathione-*S*-transferase; YEATS2, YEATS domain-containing 2; SUMO, small ubiquitin-like modifier; GA, ginkgolic acid.

Based on our SUMO-SIM model, YEATS2 is potentially SUMOylated. To ascertain the possibility, we first assessed the interaction between YEATS2 and Ubc9. Endogenous Ubc9 and OGA were detected to form a complex (Fig. 5*A*). Moreover, we observed the interaction between overexpressed proteins (Fig. 5*B*). Recombinant GST-Ubc9 proteins could pulldown HA-YEATS2 (Fig. 5*C*). And overexpression of HA-Ubc9 in HEK-293T cells significantly attenuated endogenous YEATS2 levels (Fig. 5*D*). These results are in line with our model that YEATS2 is SUMOylated, which targets it to CMA.

We observed that both endogenous and overproduced YEATS2 is SUMOylated (Fig. 5, *E* and *G*). Then, cells were cotransfected with HA-YEATS2, Flag-Ubc9, and Myc-SUMO1 plasmids. Cellular lysates were IPed, and the immunoprecipitates were subject to MS analysis, which revealed that YEATS2 K592 was modified by SUMO (Fig. 5*F*). The K592R mutant significantly reduced SUMOylation of YEATS2 (Fig. 5*G*). We also validated the specificity of the SUMO band by GA (Fig. 5*H*). And YEATS2-K592 is conserved in various organisms (Fig. 5*I*). Taken together, we generated a potential list of CMA client proteins, which are highly prone to SUMOylation and subsequent SUMOylation-SIM targeting to CMA.

### SUMOylation targets YEATS2 to CMA

**Figure 6:**
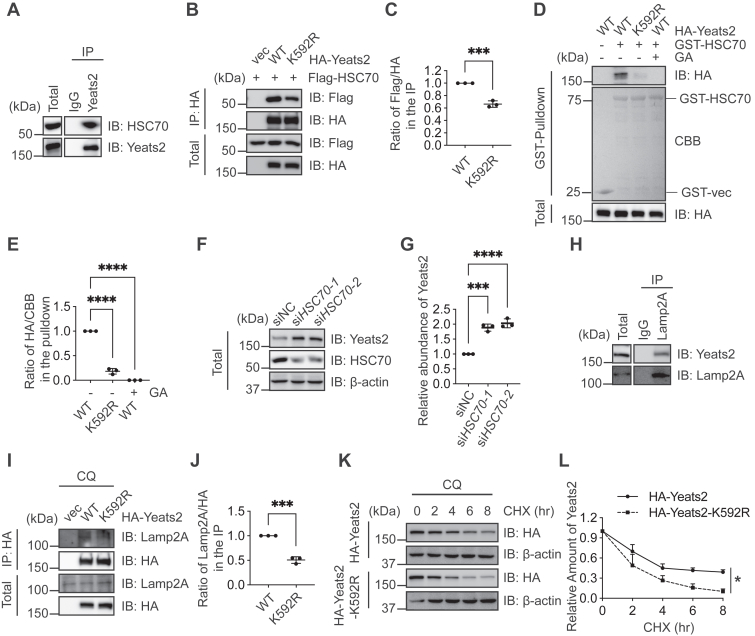
**SUMOylation targets YEATS2 to CMA.***A,* co-IP between endogenous YEATS2 and HSC70. *B,* cells were transfected with HA-YEATS2-WT, HA-YEATS2-K592R, and Flag-HSC70 plasmids. *C*, the quantitation of (*B*). *D*, cells were transfected or treated with 100 μM GA for 24 h, as indicated. Recombinant GST-HSC70 proteins were incubated with the cellular lysates, and GST pull-down experiments were carried out. *E*, the quantitation of (*D*). *F*, cells were transfected with two siRNAs targeting *HSC70*. The cellular lysates were collected and subject to IB assays. *G*, the quantitation of (*F*). *H*, co-IP between endogenous Lamp2a and YEATS2. *I*, HEK-293T cells were transfected with HA-vec, HA-YEATS2-WT, and HA-YEATS2-K592R plasmids and treated with 10 μM CQ for 3 h. *J*, the quantitation of (*I*). *K*, CHX pulse-chase assays. Cells were transfected with HA-YEATS2-WT or K592R plasmids, and then treated with CQ and CHX for different durations. The quantitation in (*E*), (*G*), and (*L*) was carried out with one-way ANOVA, in(*C*) and (*J*) were carried out with Student's *t* test. ∗*p* < 0.05; ∗∗∗*p* < 0.001; and ∗∗∗∗*p* < 0.0001. Lamp2a, lysosome-associated membrane protein type 2a; OGA, O-GlcNAcase; GST, glutathione-*S*-transferase; GA, ginkgolic acid; IB, immunoblotting; YEATS2, YEATS domain-containing 2; CQ, chloroquine; HSC70, heat shock cognate protein 70; CHX, cycloheximide; CMA, chaperone-mediated autophagy.

Next, we sought to determine whether SUMOylation targets YEATS2 to CMA. We first found that endogenous YEATS2 and HSC70 form a complex (Fig. 6*A*). We hypothesize that some client proteins modified by SUMOylation are likely to be degraded by the CMA pathway, since the SUMO consensus motifs (ψKXE) bears some resemblance to the KFERQ motif. We examined the interaction between the SUMO mutation K592R of YEATS2 and HSC70 and found that the affinity was weakened (Fig. 6, *B* and *C*). Similarly, the pulldown between HA-YEATS2-K592R and recombinant GST-HSC70 proteins also decreased (Fig. 6, *D* and *E*). We treated the cells with GA, and the pulldown of HA-YEATS2 and GST-HSC70 was completely abolished (Fig. 6, *D* and *E*). Moreover, we knocked down HSC70 with two independent si*HSC70* sequences, and YEATS2 accumulated in these cells (Fig. 6, *F* and *G*). YEATS2-K592R also shows less association with Lamp2a (Fig. 6, *I* and *J*) and CQ + CHX assays demonstrate that YEATS2-K592R is less stable (Fig. 6, *K* and *L*). These results indicate that SUMOylation targets YEATS2 to CMA.

## Discussion

**Figure 7:**
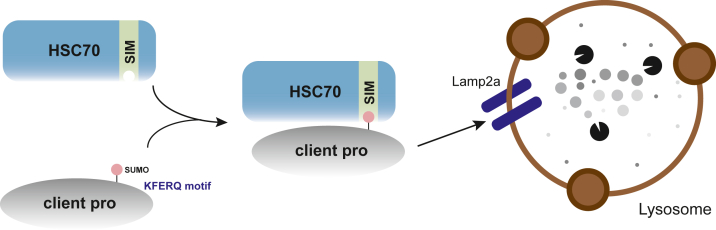
**Working model.** We propose a model in which a subset of client proteins amenable to CMA is SUMOylated. SUMOylated clients bind the SUMO-interacting motif (SIM) of Hsc70, thereby facilitating the association between CMA clients and Hsc70 the chaperone. The KFERQ motif further enhances this association. Together, the KFERQ motif and the SUMO-SIM binding deliver the client to Lamp2a the receptor for CMA in the lysosome. SUMO, small ubiquitin-like modifier; CMA, chaperone-mediated autophagy; HSC70, heat shock cognate protein 70; Lamp2a, lysosome-associated membrane protein type 2a.

In this work, we first showed that OGA is SUMOylated at K358. We further demonstrated that SUMOylation targets OGA to CMA *via* the SIM in the CMA chaperone Hsc70. We then found that the SUMO-SIM binding also applies to other CMA substrate proteins, such as the newly identified CMA client YEATS2, as revealed in our label-free quantitative MS (Fig. 7). Thus, alongside the well-known KFERQ motif, we identified a new SUMO-code in CMA: SUMOylated clients bind to HSC70 SIM.

PTMs, such as phosphorylation, ubiquitination, and acetylation, have been shown to play pivotal roles in autophagy (19). PKM2, for instance, is acetylated at K305, which shunts PKM2 to CMA (18). Intriguingly, PKM2 is also SUMOylated at K270 (17). In light of our data, it is possible that SUMOylation (perhaps at other residues) may also target PKM2 to CMA.

YEATS2 is a histone reader that binds Histone H3K27ac (20), crotonylation on lysine 27 (H3K27cr) (21) and lysine benzoylation (Kbz) (22). In non–small cell lung cancer, YEATS2 is amplified. It recruits the Ada-two-A–containing complex to the promoters of H3K27ac-containing active genes and generates H3K9ac that contributes to tumorigenesis (20). Therefore, therapeutic potential is currently being explored by developing YEATS domain inhibitors targeting π-π-π stacking in the YEATS–Kcr complex (23). Our work not only revealed that YEATS2 is SUMOylated at K592, but also showed that YEATS2 is degraded *via* the CMA pathway, which could be further utilized for therapeutic purposes.

Our proteome profiling work revealed many potential CMA targets that were previously identified to be SUMOylated, for instance, the transcription repressor Yin Yang 1 (SUMOylated (24)), the transcriptional regulatory protein Bcl11b (SUMOylated (25)), and the nucleoporin Nup107 (associating with the SUMO-specific isopeptidase SENP2 (26)). We believe our work thus would expand the CMA client protein pool and provide more valuable insights into the functionality of CMA.

Our work also shows that OGA is shunted to CMA-mediated degradation. As OGT is known to have pleiotropic substrates, chemical biology tools have been channeled into studying OGA in an effort to explore its therapeutic potential. A nanobody-fused split OGA has been instrumented as an O-GlcNAc eraser for selective deglycosylation (27). OGA has also been fused with an intein triggered by 4-hydroxytamoxifen to induce its deglycosidase activity in a time- and dosage-dependent manner (28). Our work revealed new mechanisms that regulate OGA abundance and may have the potential to be exploited for new chemical tool development.

## Experimental procedures

### Cell culture, antibodies, and plasmids

HEK293T cells were purchased from American Type Culture Collection. The cell lines were validated using short tandem repeat profiling and free from *mycoplasma* contamination for all experiments. Antibodies were as follows: anti-Flag (Sigma, F1804), anti-Myc (PTM BIO, PTM-5390), anti-HA (Abmart, 26D11), anti-IgG (Sigma, R2665), anti-OGA (Abcam, ab124807), anti-SUMO1 (Invitrogen, 33-2400), anti-SUMO2 (Immunoway, YN0072), anti-HSC70 (Abcam, ab19136), anti-Lamp2A (Abcam, ab18528), anti-Ubc9 (Abcam, ab33044), anti-β-actin (Immunoway, YM3028), and anti-YEATS2 (Proteintech, 24717-1-AP). si*HSC70* UTR: GAACAAGAGAGCTGTAAGA, GTGCCATGACAAAGGATAA.

### Immunoprecipitation and immunoblotting

Immunoprecipitation and immunoblotting experiments were performed as described before (29). Cells were harvested and washed twice with PBS. Collected cells were lysed with 200 mM lysis buffer (200 mM NaCl, 1 mM EDTA, 20 mM Tris (pH 7.5), and 0.5% NP-40) containing a protease inhibitor cocktail (Roche). For the SUMOylation assay, 20 mM NEM was also added.

The following primary antibodies were used for immunoblotting: anti-Flag (1:4000), anti-HA (1:60,000), anti-Myc (1:4000), anti-OGA (1:1000), anti-SUMO1 (1:1000), anti-SUMO2 (1:1000), anti-HSC70 (1:4000), anti-Lamp2A (1:1000), anti-Ubc9 (1:2000), anti-β-actin (1:5000), and anti-YEATS2 (1:1000). LAS-4000 was employed to detect signals and quantitated by the Multi Gauge software (Fujifilm) (www.fujifilm.com/us/en).

### Chemicals

GA (MedChemExpress, HY-N0077) was prepared at 100 mM with dimethyl sulfoxide (DMSO), and then the cells were treated with 100 μM for 24 h. CQ (MCE, HY-17589A) was prepared at 50 mM with DMSO, and then the cells were treated with 10 μM for 3 h. CHX (SIGMA, C7698-5G) was prepared at 20 mg/ml with DMSO, and then the cells were treated with 150 μg/ml for different times. N-Ethylmaleimide (NEM) (Solarbio, N8760) was prepared at 2 M with DMSO, and then the cells were treated with 20 mM NEM to examine SUMOylation.

### MS to identify SUMOylation sites

For the identification of SUMOylation by MS, proteins isolated by gel electrophoresis were digested with trypsin (Promega) and Glu-C (Promega) in 100 mM NH_4_HCO_3_ pH 8. The LC-MS/MS analysis was performed on an Easy-nLC 1000 II HPLC (Thermo Fisher Scientific) coupled to a Q-Exactive HF mass spectrometer (Thermo Fisher Scientific). Peptides were loaded on a precolumn (100 μm ID, 6 cm long, packed with ODS-AQ 10 μm, 120 Å beads from YMC Co., Ltd) and further separated on an analytical column (75 μm ID, 15 cm long, packed with Luna C18 1.9 μm 100 Å resin from Welch Materials) using a linear gradient from 100% buffer A (0.1% formic acid in H_2_O) to 30% buffer B (0.1% formic acid in acetonitrile), 70% buffer A in 70 min at a flow rate of 320 nl/min. The top 20 most intense precursor ions from each full scan (resolution 60,000) were isolated for higher-energy collisional dissociation (HCD) MS2 (resolution 30,000; normalized collision energy 27) with a dynamic exclusion time of 30 s. Precursors with a charge state of 1+, 7+ or above, or unassigned, were excluded.

The software pFind3 (30, 31) was used to identify SUMOylated peptides by setting a variable modification of 343.1491 Da at K. The mass accuracy of precursor ions and that of fragment ions were both set at 20 ppm. The results were filtered by applying a 1% false discovery rate cutoff at the peptide level and a minimum of one spectrum per peptide. The MS2 spectra were annotated using pLabel (32).

### Label-free quantitative MS

#### Sample preparation

The protein precipitates were incubated with 6 M urea, 10 mM DTT in 50 mM ammonium bicarbonate at 37 ^°^C for 45 min, and then incubated in 10 mM iodoacetamide in 50 mM ammonium bicarbonate at ambient temperature for 1 h in the dark. The solution was then diluted to a urea concentration of 2 M using 50 mM ammonium bicarbonate, followed by trypsin digestion with an enzyme:protein ratio of 1:40 at 37 °C overnight. Formic acid was added to the solution to a final concentration of 0.1% to quench the digestion. All samples were vacuum-centrifuged to dryness and resuspended in 0.1% formic acid in water prior to LC-MS/MS analysis.

#### LC-MS/MS parameters

Peptides were separated using a loading column (100 μm × 2 cm) and a C18 separating capillary column (75 μm × 15 cm) packed in-house with Luna 3 μm C18(2) bulk packing material (Phenomenex). The mobile phases (A: water with 0.1% formic acid and B: 80% acetonitrile with 0.1% formic acid) were driven and controlled by a Dionex Ultimate 3000 reversed phase liquid chromatography nano system (Thermo Fisher Scientific). The liquid chromatography gradient was held at 2% for the first 8 min of the analysis, followed by an increase from 2% to 10% B from 8 to 9 min, an increase from 10% to 44% B from 9 to 63 min, and an increase from 44% to 99% B from 63 to 68 min.

For the samples analyzed by Orbitrap Fusion LUMOS Tribrid Mass Spectrometer, the precursors were ionized using an EASY-Spray ionization source (Thermo Fisher Scientific) source held at +2.0 kV compared to ground, and the inlet capillary temperature was held at 320 °C. Survey scans of peptide precursors were collected in the Orbitrap from 350 to 1600 Th with an automatic gain control target of 400,000, a maximum injection time of 50 ms, radio frequency lens at 30%, and a resolution of 60,000 at 200 *m/z*. Monoisotopic precursor selection was enabled for peptide isotopic distributions, precursors of z = 2 to 7 were selected for data-dependent tandem mass spectrometry (MS/MS) scans for 3 s of cycle time, and dynamic exclusion was set to 15 s with a ±10 ppm window set around the precursor monoisotope.

In HCD scans, an automated scan range determination was enabled. An isolation window of 1.6 Th was used to select precursor ions with the quadrupole. Product ions were collected in the Orbitrap with the first mass of 110 Th, an automatic gain control target of 50,000, a maximum injection time of 30 ms, HCD collision energy at 30%, and a resolution of 15,000.

### Data analysis

Data processing was carried out using Thermo Proteome Discoverer 2.4 using a SwissProt Human Database (version 2017-10-25). Carbamidomethyl (Cys) were chosen as static modification, and oxidation (Met) was chosen as variable modification. Mass tolerance was 10 ppm for precursor ions and 0.02 Da for fragment ions. Maximum missed cleavages were set as 2. Peptide spectral matches were validated using the Percolator algorithm, based on q values at a 1% false discovery rate. For label-free quantitation, protein abundances were calculated by summing sample abundances of the corresponding peptides.

## Data availability

The mass spectrometry proteomics data have been deposited to the ProteomeXchange Consortium (https://proteomecentral.proteomexchange.org) *via* the iProX partner repository (33, 34) with the dataset identifier PXD056772.

## Supporting information

This article contains supporting information.

## Conflict of interest

The authors declare that they have no conflicts of interest with the contents of this article.
