# Supplementary material for: SUMOylation targets O-GlcNAcase to chaperone-mediated autophagy
Source: J Biol Chem. 2025 May 29;301(7):110314. doi: 10.1016/j.jbc.2025.110314 (PMC12240075; doi:10.1016/j.jbc.2025.110314)
Supplement: Figure S1 [file mmc2.pdf]

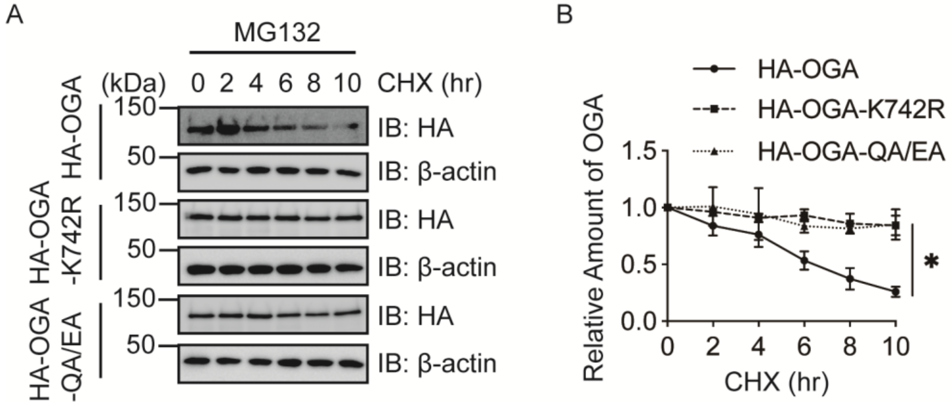

Fig. S1. *A*, cycloheximide (CHX) pulse-chase assays. Cells were transfected with HA-OGA-WT, -K358R or -Q356A/E360A plasmids, then treated with MG132 and CHX for different durations. *B*, the quantitation of (*A*). Quantitation in (*B*) was carried out with one-way ANOVA. * $p < 0.05$.
